# Cell Patterns Emerge from Coupled Chemical and Physical Fields with Cell Proliferation Dynamics: The *Arabidopsis thaliana* Root as a Study System

**DOI:** 10.1371/journal.pcbi.1003026

**Published:** 2013-05-02

**Authors:** Rafael A. Barrio, José Roberto Romero-Arias, Marco A. Noguez, Eugenio Azpeitia, Elizabeth Ortiz-Gutiérrez, Valeria Hernández-Hernández, Yuriria Cortes-Poza, Elena R. Álvarez-Buylla

**Affiliations:** 1 Instituto de Física, Universidad Nacional Autónoma de México (UNAM), México, Distrito Federal, México; 2 Universidad Autónoma de la Ciudad de México, Mexico, Distrito Federal, México; 3 Instituto de Ecología, Universidad Nacional Autónoma de México, México, Distrito Federal, México; 4 Centro de Ciencias de la Complejidad-C3, Universidad Nacional Autónoma de México, Distrito Federal, México; Northeastern University, United States of America

## Abstract

A central issue in developmental biology is to uncover the mechanisms by which stem cells maintain their capacity to regenerate, yet at the same time produce daughter cells that differentiate and attain their ultimate fate as a functional part of a tissue or an organ. In this paper we propose that, during development, cells within growing organs obtain positional information from a macroscopic physical field that is produced in space while cells are proliferating. This dynamical interaction triggers and responds to chemical and genetic processes that are specific to each biological system. We chose the root apical meristem of *Arabidopsis thaliana* to develop our dynamical model because this system is well studied at the molecular, genetic and cellular levels and has the key traits of multicellular stem-cell niches. We built a dynamical model that couples fundamental molecular mechanisms of the cell cycle to a tension physical field and to auxin dynamics, both of which are known to play a role in root development. We perform extensive numerical calculations that allow for quantitative comparison with experimental measurements that consider the cellular patterns at the root tip. Our model recovers, as an emergent pattern, the transition from proliferative to transition and elongation domains, characteristic of stem-cell niches in multicellular organisms. In addition, we successfully predict altered cellular patterns that are expected under various applied auxin treatments or modified physical growth conditions. Our modeling platform may be extended to explicitly consider gene regulatory networks or to treat other developmental systems.

## Author Summary

The emergence of tumors results from altered cell differentiation and proliferation during organ and tissue development. Understanding how such altered or normal patterns are established is still a challenge. Molecular genetic approaches to understanding pattern formation have searched for key central genetic controllers. However, biological patterns emerge as a consequence of coupled complex genetic and non-genetic sub-systems operating at various spatial and temporal scales and levels of organization. We present a two-dimensional model and simulation benchmark that considers the integrated dynamics of physical and chemical fields that result from cell proliferation. We aim at understanding how the cellular patterns of stem-cell niches emerge. In these, organizer cells with very low rates of proliferation are surrounded by stem cells with slightly higher proliferation rates that transit to a domain of active proliferation and then of elongation and differentiation. We quantified such cellular patterns in the *Arabidopsis thaliana* root to test our theoretical propositions. The results of our simulations closely mimic observed root cellular patterns, thus providing a proof of principle that coupled physical fields and chemical processes under active cell proliferation give rise to stem-cell patterns. Our framework may be extended to other developmental systems and to consider gene regulatory networks.

## Introduction

The study of stem-cell niche patterns, and specifically how stem cells can maintain their totipotent state while simultaneously giving rise to daughter cells that obtain distinct fates to form differentiated tissues and organs, is fundamental to understanding the development of multicellular organisms [1]. Although plants and animals have key differences in their development (e.g. lack of cell migration in plant development), the cellular organization of stem-cell niches in both lineages reveals striking similarities [1], [2]. In both plants and animals, stem-cell niches are formed by an organizer group of cells with low rates of division, surrounded by stem cells with slightly higher division rates. Moving distally from the organizer and stem cells, cells proliferate at high rates. This proliferation domain (also called amplification domain) is bordered by the elongation and then the differentiation domains where proliferation stops and expansion and differentiation, respectively, take place [1], [3].

Gene interactions within intracellular complex regulatory networks (GRN) [4], [5] or from morphogen dynamics at supracellular scales (see [6], [7]) are fundamental for proper growth and development. Indeed organ and tissue development, as well as stem cell maintenance relies to a great extent on complex transcriptional regulatory networks and chemical fields. However, these are not the only components of pattern formation. It is now recognized that physical fields are also critical to explain developmental patterns, as they may provide positional information that modifies cell behavior and differentiation (see [6], [7]). At the cellular level, the simplest physical constraint is space. Cell expansion is driven by turgidity, which is an important force acting on the cell wall [8]. The cell wall is a network of rigid cellulose microfibrils cross-linked by polysaccharides and proteins, that confer stiffness to the wall and allows it to resist turgidity [9]. Expansion of the cell is opposed by the rigidity of the cell wall, producing a real stress field. Recent evidence shows that these kind of mechanical cues are transmitted to the nucleus and, directly or indirectly, regulate transcription factors (see for instance [10] and references therein).

Given the complexity of the processes involved in the coupling of developmental restrictions, mathematical and computational tools have become indispensable in our efforts to understand the network of interactions involved in cellular differentiation and organ development. Previously [11] we demonstrated that a simplified version of the originally proposed GRN [12], [13] involved in floral development, could be coupled with a mesoscopic physical field. This provides positional information to cells in the floral meristem which is required to produce the overall spatial pattern of cells observed during early flower development. This and other similar studies [14] suggest that robust morphogenetic patterns in multicellular organisms emerge from complex interconnected dynamical processes, acting at different levels of organization and spatio-temporal scales. However, models that include such dynamical processes into the dynamics of pattern formation in multicellular organs are in their infancy [15], [16]. Here we use the *Arabidopsis thaliana* (*A. thaliana*) root apical meristem as a study system to propose a model that couples cell proliferation and growth with chemical-physical dynamical processes to predict the emergence of patterns in a multicellular and multi-scale system.

**Figure 1:**
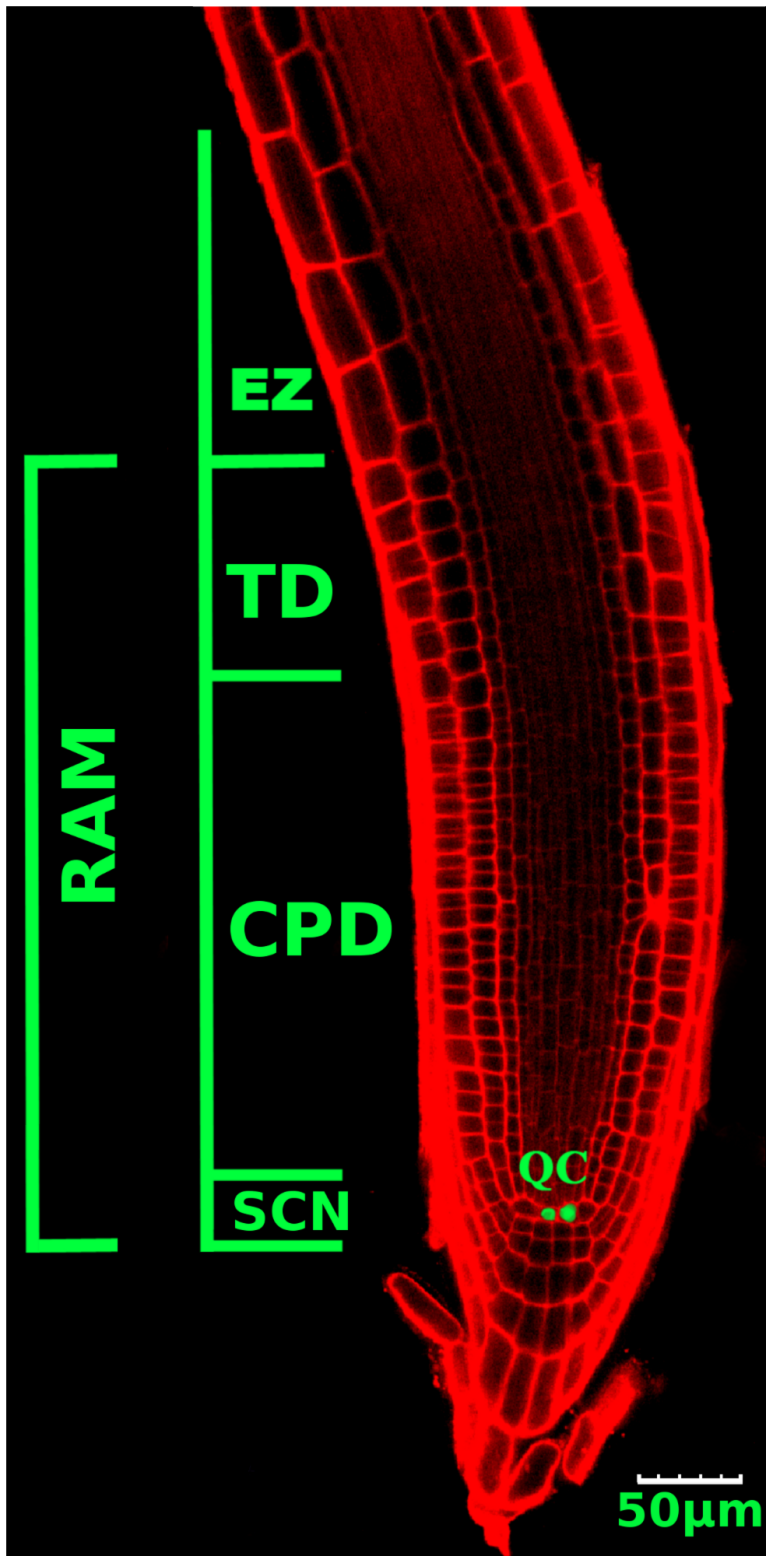
Confocal microscopy image of the *A. thaliana* root tip. The stem-cell niche (SCN) with the quiescent cells (QC, in green) and surrounding stem cells, the cell proliferation domain (CPD) with actively proliferating cells, the transition domain (TD) and the elongation zone (EZ), where cells do not proliferate, are indicated. The SCN, CPD and TD comprise the RAM.

The *A. thaliana* root has become an important experimental model for understanding the molecular, cellular and biophysical basis of morphogenesis in complex organs. This is due to its relatively simple cellular structure and its indeterminate growth, which gives rise to a multicellular structure with distinct cell proliferation and elongation domains. Importantly, the root apical meristem exhibits the typical cellular organization of stem cells described above (see Fig. 1). At the tip of roots stem cells are located surrounding the quiescent centre cells or the organizer cells (green cells in Fig. 1); together, they constitute the stem-cell niche (SCN) of the Arabidopsis root. Towards the base of the plant, the stem cells transit to a cell proliferation domain (CPD) where cells have high rates of cell division (also called proximal meristem by some authors, for example: [17]), then they enter a transition domain (TD), where cells have low or no probability of dividing, but they have not started to elongate [18]. The SCN, the CPD and the TD comprise the root apical meristem (RAM). More distally from the organizer center, cells cease to proliferate and start to grow in the elongation domain (EZ). Upon expanding to their maximum length, cells attain their final fate in the differentiation domain and produce the different tissues of the root.

**Figure 2:**
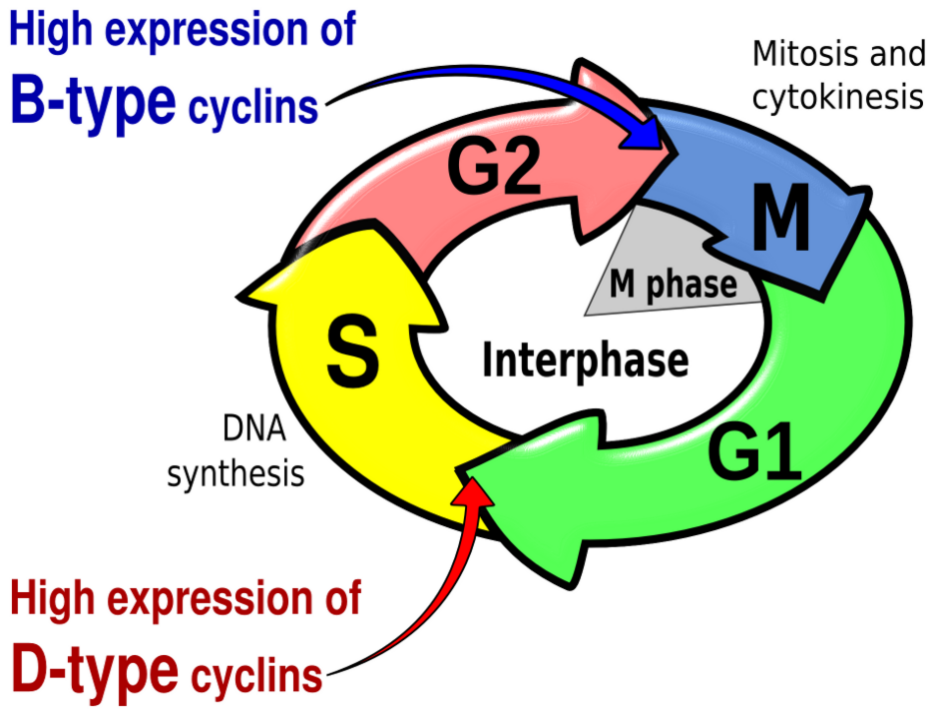
Simplified scheme of the cell cycle. The four main phases and the expression of two key cyclins are indicated.

Key experimental data on cell cycle regulation and auxin behavior in the root are used to develop our model. Patterns of cell proliferation along the root longitudinal (apical-basal) axis are greatly affected by the dynamics of the cell cycle itself and by the concentration of several plant hormones, including auxin [19]–[23]. Cells in the root proliferation domain of the RAM undergo several rounds of division before starting to elongate in the elongation domain. A complex network of regulatory interactions controls the cell cycle, in which cyclin proteins are key regulators. As their name suggests, the expression of cyclins oscillates during each cell cycle. At the beginning of each cell cycle, D-type cyclins (CYCD) induce the expression of the RETINOBLASTOMA-RELATED (RBR) gene through E2F-RBR pathway. RBR is a negative regulator of E2F transcription factors, which activate the transcription of mitotic cyclin CYCB. Later, CYCB cyclins are degraded by the Anaphase-promoting complex/cyclosome, thus completing the cycle and returning to the beginning of the cell cycle (see reviews in: [24], [25]). For the present study, the oscillatory and time differential expressions of CYCD and CYCB are sufficient to represent the cell cycle dynamics. The cell cycle phases and main regulators are illustrated in Fig. 2.

Auxin is a phytohormone involved in almost every aspect of plant development (see [26]–[32]). Auxin is a key regulator of cell proliferation and cell elongation, and also modulates cell cycle progression and cyclins [33]–[35]. Auxin has been shown to upregulate mitotic cyclin (CYCA and CYCB) expression, and the over-expression of CYCA can partially recover the phenotype caused by low auxin levels, thus suggesting that auxin promotes cell cycle progression [35]. It is also well-documented that auxin gradients correlate with apical-basal patterns of cell proliferation and elongation along the root (see [35]–[41]). There is an auxin concentration gradient along the longitudinal axis of the root, with the maximum concentration detected at the stem-cell niche, specifically in the quiescent center [41], [42]. While other hormones are important in root growth and development, we exclusively consider auxin due to its clear role in regulating cell cycle dynamics and its measurable concentration gradient that correlates with root developmental patterning [26].

Theoretical and experimental studies suggest that such auxin gradients depend critically on the polar localization of the auxin efflux transporter proteins, belonging to the *PINFORMED* gene family (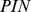) (see [43]–[47]). Five 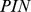 members are expressed throughout the root, namely *PIN1*, *2*, *3*, *4* and *7*. The proteins PIN1, 3, 4, and 7 maintain a continuous auxin flow from the base to the apex along the central tissues of the root. At the most apical zone, below the QC, auxin is laterally redistributed to the peripheral tissues by PIN3, 4, and 7. Finally, PIN2 directs flow from the root apex to the base in addition to lateral auxin flow in peripheral tissues. In conjunction, all PIN proteins create a reverse fountain mechanism that maintains an auxin gradient along the root [43], [46], [48].

Physical signals have been shown to affect auxin distribution, for instance auxin gradients can be modified by mechanically-induced root bending [49], [50], or by changes in gravitational fields [51], [52]. Polar auxin transport and microtubule orientation also respond to mechanical forces in the shoot apical meristem [53], [54]. Such evidence suggests that auxin transport is affected by and tightly coupled to physical forces. Furthermore, there is increasing evidence that mechanical stress is extremely important for plant morphogenesis; for instance, experiments show that differentiation of mesenchymal cells is influenced by the rigidity of the intracellular matrix [55].

In this paper we propose a simple model to study the interaction between cell proliferation dynamics, local auxin concentration (that in turn depends on the polar localization of PIN transporters in the cell membranes), and an elastic physical field arising from the inherent growth dynamics of the root. Our model provides a formal tool that can be used to understand and predict the emergence of the cellular patterns in the root tip. This type of model can be extended to explore similarities in stem-cell niche organization and subsequent cellular behaviors (proliferation, elongation and differentiation) of plants and animals, and to predict if such cellular organization might be explained by the coupling of generic non-linear physical and chemical fields relevant to cell proliferation dynamics. Our model is validated with experimental measurements on cell size and proliferation patterns along *A. thaliana* root, and sets the stage for developing similar approaches in other systems.

## Model

Roots are three-dimensional structures. However, the root tip presents a consistent cylindrical symmetry that allows one to ignore changes in the transverse plane of the root when considering growth models. It is therefore possible to use a two-dimensional domain consisting of undifferentiated cells to represent the shape of the root tip. This approximation allows for numerical analysis of the model in 2D space. The model can be validated by comparing the patterns obtained with those observed experimentally in longitudinal histological or optical sections as the ones readily used in experimental assays done with *A. thaliana* roots. In some cases we have also compared our results obtained from 3D roots.

**Figure 3:**
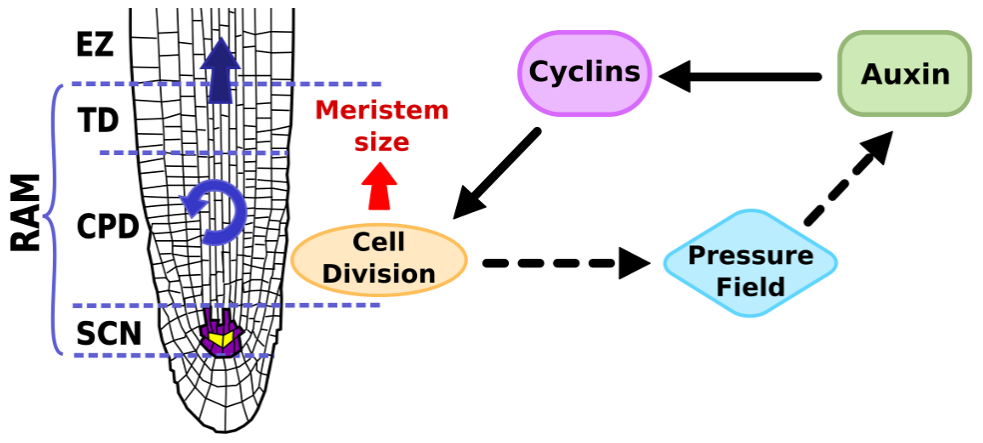
Histological drawing of the *A. thaliana* root tip. Here we show the SCN and the same domains as shown in Fig. 1 are indicated along the root apical-basal axis, as well as an schematic representation of the processes that are included in the cellular model and their interactions.

Based on the shape and spatial arrangement of the root, we conclude that cell reproduction in the early stages of root development involves mainly three chained dynamics of cell proliferation and resulting elastic field, and of the pattern of auxin concentration, whose co-occurrence provides the spatial information necessary to regulate the proliferation rate of each cell and to ultimately determine its future fate during differentiation (see Fig. 3 for a schematic summary of the processes to be modeled and the region of the root in which they take place).

Our first hypothesis is that a macroscopic physical field along the root tip results from cell growth and proliferation within this tissue in addition to constrained conditions imposed by the root cap and the epidermal cells surrounding the root. We propose that this field is elastic in nature and can be characterized by point functions of stress, pressure, or local mechanical forces that stem from the symplastic nature of plant tissues that are formed by interconnected cells surrounded by cellulose cell walls. Perfect equilibrium represents a state in which there are no mechanical forces acting anywhere in the system. In practice, this equilibrium cannot be completely achieved because of the geometric hindrances that impede the macroscopic system to reach a global minimum in the energy landscape, trapping it in a local minimum. In this situation there are remnant forces, and consequently the field is not uniform. Our model considers this lack of uniformity as a source of spatial information.

Our second hypothesis is that the synthesis, degradation and transportation of auxin respond to the local elastic field in a direct way, producing a dynamic pattern of auxin concentration along the longitudinal axis of the root tip. This is important, since the dynamical behavior of the formation of an auxin gradient should be very different from the relaxation dynamics of the elastic field, and it should occur at a different spatio-temporal scale.

Our third hypothesis proposes a direct relationship between auxin concentration and cell cycle regulation that determines cell proliferation rate. In the locations where cells divide and expand, the elastic field is greatly modified and, in fact, it is reinforced locally. This, in turn, affects the cell proliferation dynamics.

In short, we propose that the interaction among three different coupled dynamics (the relaxation of the physical field, the transport and concentration gradient of auxins and the oscillations of the cell cycle regulators, i.e., the cyclins) capture the key aspects underlying the overall emergent patterns of cell proliferation/elongation, as well as the macroscopic appearance and overall shape of the root. Our model includes the three dynamical processes (cell proliferation, auxin spatio-temporal concentration patterns and the elastic field) and their couplings in a two-dimensional domain that represents a longitudinal section of the root.

### Cell dynamics and physical fields

We start by modeling the space occupied by a cell. Expansion of the cell volume, whether by turgidity or growth, is opposed by the rigidity of the cell wall producing a real stress field [9], [56]. This field is also present at the larger scale of a group of cells, such as within the root apical meristem, since the increase in volume required by cell growth and division is opposed by the surface forces exerted by the root cap and epidermal cells surrounding it [57]. From this perspective, it is logical to assume that this stress field is self-regulated, that is, the accumulation of local stress (or pressure) triggers mechanisms that prevent (or enhance) cell division and growth. This assumption of self-regulation has been incorporated into previous models of cellular interactions: Dupuy and collaborators [58] used a rigidity matrix to model the relationship between cell displacement and implied forces. A form of potential energy has likewise been proposed as a way of describing the equilibrium between turgor and cell wall resistance [59]. Finally, in a recent paper investigating the floral meristem of *A. thaliana*, potential energy was proposed as the means of regulating auxin transport [15].

In our model for the root apex, we define a spatial domain in which a potential function acts. The spatial derivatives of this function render the mechanical force as a function of time and space. Taking advantage of the radial symmetry of the region of the root tip, we consider a two-dimensional space and divide it into cells. We simulate cells by a Voronoi diagram obtained from a collection of generating points that represent the position assigned to each cell.

### Voronoi diagrams

A Voronoi diagram, or tessellation, associated with a collection of points assigns to each point a limited region of space in the form of a convex polygon (polyhedron in three dimensions). Voronoi cells are used nowadays in many fields of science, however it was Honda [60] who first proposed the use of 2D Voronoi to model cells in a biological context.

Our domain is defined as follows: 1) We construct a regular shape with points on a rectangle and a parabolic tip. The exterior points are fixed and represent the epidermal cells surrounding the ground tissue of the root (See Videos S1 and S2). 2) These points in the border cannot define a convex polygon, so the corresponding cells have a point at infinity. 3) We create 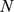 points with random coordinates in the interior of this domain and perform a Voronoi tessellation using a Delaunay triangulation algorithm.

**Figure 4:**
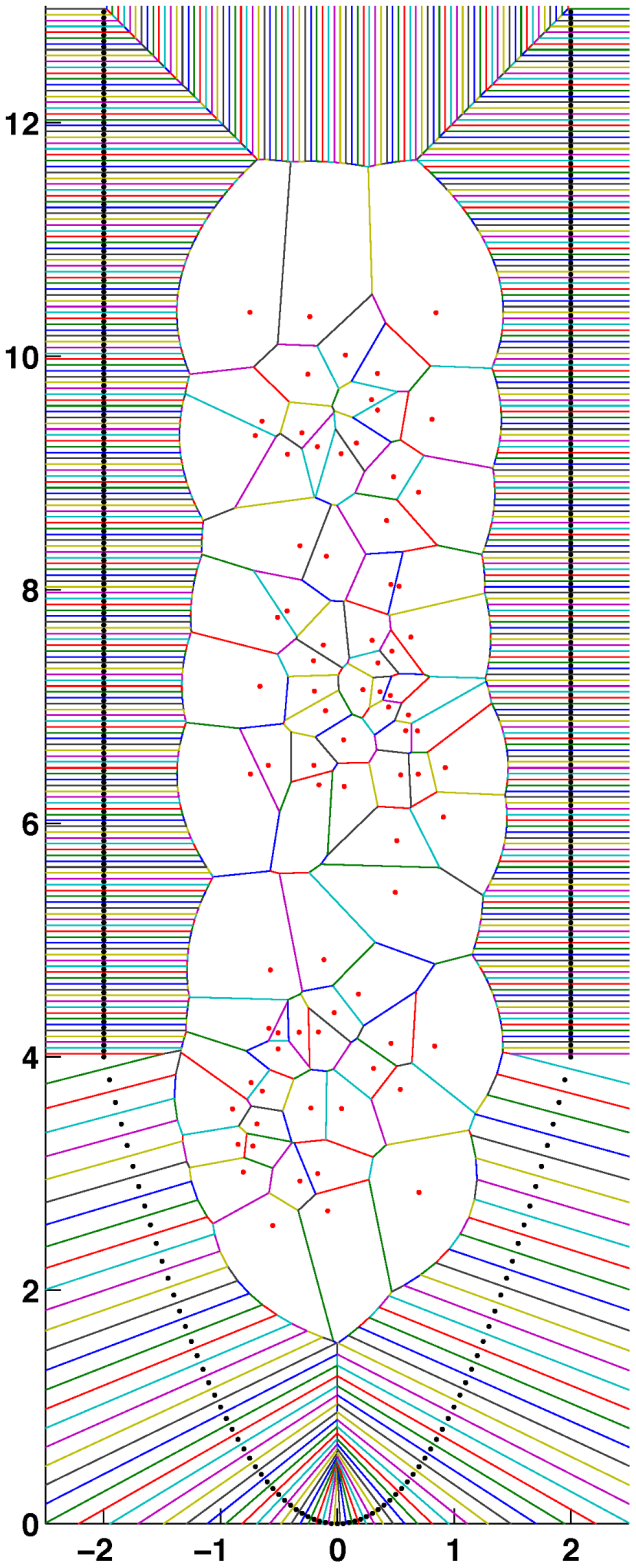
Typical initial configuration of cells after the Voronoi tessellation of random generating points.

A typical configuration is shown in Fig. 4. Observe that the areas of the cells (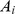) vary in size and shape, and that the generating points shown in the figure (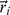) do not correspond, in general, to the centre of mass of the cells (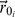).

The average 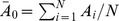 is the space that each cell would occupy in a regular hexagonal lattice. Analogously, the distance is 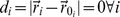 in the regular array. In two dimensions the array of cells with minimal surface energy is the hexagonal lattice, and we use this fact to define a potential function around this equilibrium configuration.

### Elastic fields

Previous studies have used springs to simulate the interactions among cells [61], and the elements of the cellular walls [62], [63]. In our case the equilibrium area 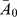 could be used to fix the size of mature cells, so deviations from this value would represent immature cells. If the cells in the tissue tend to be isotropic in shape, then a value of 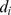 different from zero would represent cells with the wrong shape and, consequently, largely stressed.

Regardless of the actual functional form of the energy potential, it is possible to make a Taylor expansion around the equilibrium state retaining only the first non-zero terms, provided one considers small deviations from equilibrium. The first non-trivial contributions correspond to a quadratic form, whose coefficients can be interpreted as force constants.

Therefore, we propose a harmonic potential acting on each cell 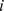


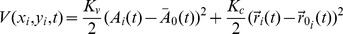
(1)where the first term tends to uniformize the size, and the second term is related with the shape of the cells. 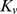 and 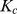 are elastic constants.

The expressions for the components of the force are:
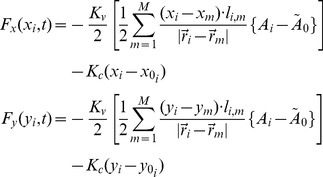
(2)where 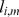 is the length of the edge shared between neighboring cells, and we have omitted the time dependence of the variables on the right hand side. All quantities in Eqs. 1 and 2 can be readily calculated with the algorithm used to define the Voronoi diagram.

Since this is a conservative system, and there is no reason to assume conservation in the root system, we include dissipation in the form of friction that simulates losses due to the inability of cells to make drastic elastic changes of shape or size. Therefore, the total force should be:
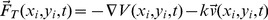
(3)where 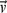 is the velocity and 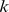 is a friction coefficient. The 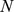 coupled dynamical equations of this newtonian system
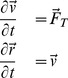
(4)can be integrated numerically by using a simple Euler method, imposing fixed boundary conditions on the fixed surface points.

**Figure 5:**
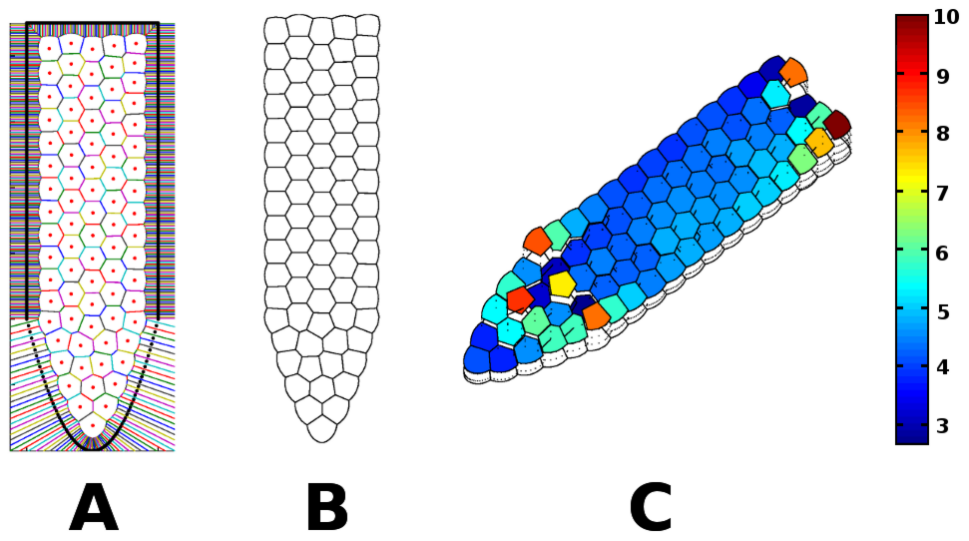
Configuration of cells after 2000 time iterations. (A) The points in Fig. 4 once they have attained equilibrium using the potential (See Video S3). (B) Final configuration of the cells in the RAM. Observe the regularity of the shapes and sizes of the cells. (C) 2D profile of the field after relaxation. Observe that it is not constant, but there are three well defined sections (See Video S4).

As an example of the relaxation process with this scheme, in Fig. 5 we show the configuration of points in Fig. 4 after 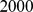 time iterations. The numerical calculation was stopped when the relative changes of the positions and velocities was less than 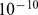. The magnitude of the constants 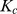, 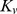 and 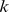 sets the units of the time variations of the dynamical behavior of the system, and should be adjusted to physical units when modeling the growth of the RAM. One should consider the number of cell divisions per unit time (2.6 events/hr), the cell production rate (between 0 and 6 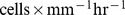) and the cell proliferation rate distribution (between 0 and 50 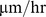) in the RAM [64]. The final form of the relaxed field suggests that it could be used to transfer positional information to the cells in the meristem. In order to achieve the latter, the auxin concentration must be coupled to the local value of the potential.

We introduced the process of cell division and proliferation into the simulation by defining two points inside a cell when it undergoes mitosis. The resulting Voronoi cells then locally alter the field, and the extra space needed for the two daughter cells is obtained by moving the upper border of the domain a proper distance to provide the exact extra space required. We show details of this process below.

### Auxin transport

It is assumed that the field 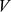 is involved in the processes of auxin transport. In any transport equation there are basically two aspects to be considered: the hydrodynamic forces compelling a fluid to move, and the diffusion phenomena. Both are important for the case of auxins. Furthermore, the process of auxin transport is recognized to be active, meaning that the transfer of matter through the cell membranes could go against the concentration gradient of auxin molecules due to the action of PIN proteins. We propose that the amount of matter 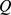 transported per unit time from cell 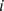 to a neighbor cell 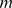 is proportional to the gradient of the field 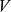:
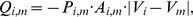
(5)where 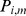 represents the permeability of the membrane and 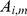 is the contact surface between the cells 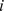 and 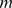 (the line 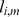 in 2D).

Observe that if the values of the 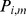
's were constant, this equation would reduce to the well known Darcy's Law in hydraulics, which is analogous to Fourier's law in heat conduction, or Ohm's law in electrical networks. However this is not the case, because of the action of the PIN proteins which are critical. Therefore, the permeability is:
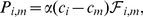
(6)where 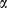 is a constant related with the time scale of the dynamics, and the direction of the flux with respect to the concentration gradient 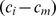 (diffusion term) is given by the logical function 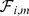. This latter function mimics the action of the PIN molecules, which attach to the membrane according to orientation and position in the domain.

We can simplify this action by considering “gates”, which could be opened (1) or closed (0) according to specified simple rules. Let 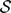 be the set of cells at the surface, i.e. in contact with the immobile epidermal cells. We have set the following rules: All gates are closed, except

1. when 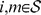 and 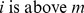,
2. or if 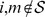 and 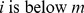,
3. or if 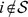 and 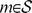 and 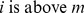.

The dynamical equation for the concentration of auxins in cell 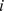 is then:
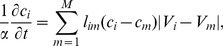
(7)where the sum is over all neighboring cells. This expression can be readily integrated numerically in parallel with Eq. 4, once the parameter 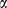 has been properly adjusted.

**Figure 6:**
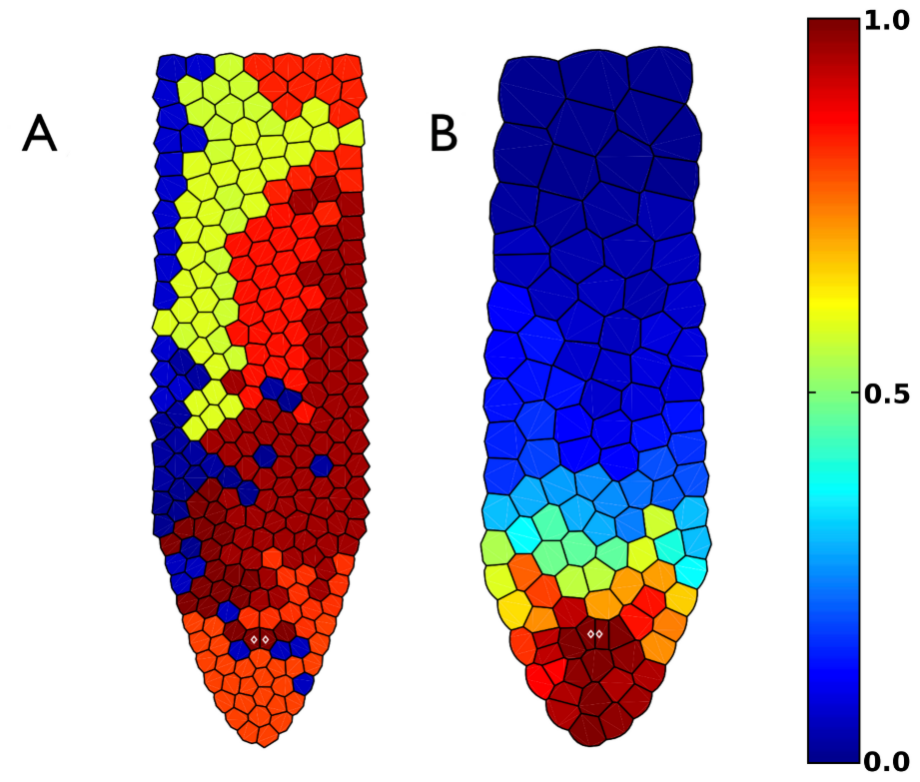
Typical numerical integration of **Eq. 7** showing the formation of auxin gradients. (A) All gates are open (no PIN action). (B) Including the logical rules to open the gates to model the PIN action.

In Fig. 6 we show the effect of the logical rules on the formation of auxin gradients. On the left we show a calculation without these rules, that is, maintaining all the membranes permeable. In (B) we incorporate the PIN action into the model. Observe that the distribution of the concentration of auxins (normalized with its maximum value) is similar to the one observed in real roots [42].

### Cell division cycle

**Figure 7:**
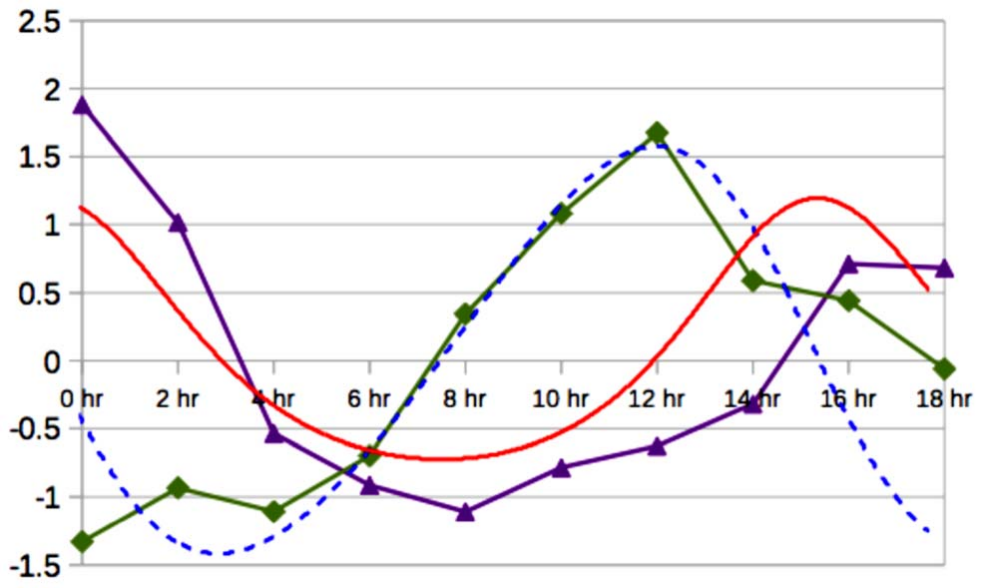
Variation of two-type cyclins concentrations and typical oscillations from the Lotka-Volterra model. Relative expression data of D-type cyclins (purple triangles) and B-type cyclins (green rhombuses) were taken from analysis of gene expression profiles using aphidicolin synchronization on Ref. [76], and are available on GENEVESTIGATOR web page. The oscillations from the Lotka-Volterra model of the inhibitor (blue dashed line) and the activator (red line) are also shown.

We shall assume that the period of the cell division cycle is regulated by the local concentration of auxins 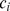. We are aware that this is an oversimplification of the complex hormonal regulation of the cell cycle in plants, but auxin has been shown to be an important component of such regulation [65]. We therefore need a model for the oscillations of cyclin concentrations. The robustness of these oscillations suggests that a non-linear oscillator would be a good model. We consider a two-component system for simplicity, considering CYCD and CYCB as the two key players. Since both undergo regular out-of phase oscillations with maxima related to the transitions between the G1-S and G2-M phases, respectively (See Fig. 2), we choose a simple Lotka-Volterra non-linear system with two components, generally used in ecology to model the predator-prey dynamics. This system presents all characteristics required for the observed time behavior of the concentration of cyclins [66]. The adimensional activator-inhibitor dynamical equations are:
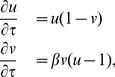
(8)where 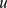 and 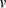 represent CYCD and CYCB, respectively. This system presents an oscillatory behavior, provided 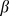 is within a certain range, whose period (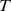) and wave shape depend only on 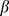 and on the boundary conditions. It is easily shown that the period is:
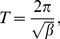
(9)which is inversely proportional to the square root of the ratio of the linear growth rate of the “prey” (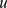) to the death rate of the “predator” (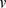). In Fig. 7 we illustrate the oscillations of both variables.

Experimental data has shown that the cell cycle is arrested if the auxin concentration is below or above certain threshold values, and that the cycle period increases with auxin concentration [65]. Therefore, we simply assume that the auxin concentration is linearly related to the only parameter of this dynamical system: 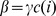. Hence, each cell has its individual clock, which runs faster or slower depending on the auxin concentration in the model under consideration. We couple this dynamical feature into the numerical calculation of the model by performing a division of cell 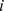 when 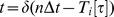 (where the 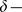
function is one when the number of iterations 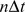, used in the Euler integration, surpasses the period). Therefore, 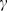 is another constant that relates the time scale of reproduction (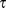) to the time step used for relaxation dynamics. Parameters 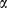 and 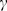 should be fitted according to the observed time scales for each of the three dynamics. Time step 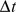 (in seconds) should be obtained as well.

In practice, the act of cellular division is performed in the following manner:

1. At each time step, advance the internal clock of all cells according to the value of the local auxin concentration given by the cell life-cycle model.
2. Detect the cells in which the internal clock completes one period (a single division event occurs every cycle), and set the clock of these cells to zero.
3. In each one of these cells, 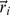 is substituted by two points, oriented at random and at equal distances of 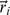. This distance is typically of the order of a quarter of the radius of the cell.
4. The kinetic energy (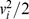) of the mother cell is equally divided between mother and daughter cells.
5. The upper boundary of the domain is shifted upwards to increase the area by the exact amount required by these new cells to grow eventually to adult size.

The changes in the domain size and the size of the new cells produce a rearrangement of all cells, and this changes the local value of the elastic field, which, at the same time, drives the auxin concentration that, in turn, regulates the division rate of all cells. We hypothesize that coupling among such three dynamics at different time scales is sufficient to produce the growth of the root with cellular patterns that mimic those of real systems in a wide region of the parameter space. We verified that the process is extremely robust against changes of initial conditions.

## Results

**Figure 8:**
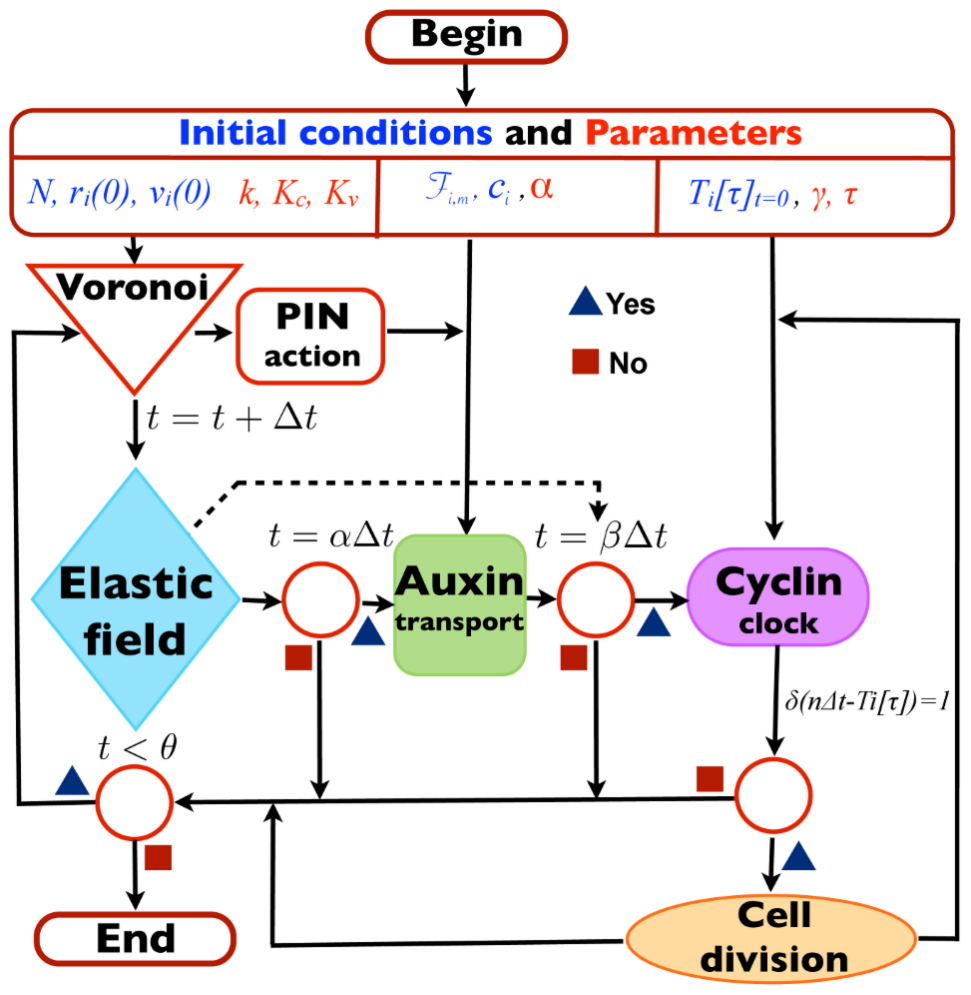
Flow-chart diagram of the program used for the numerical simulations. We show the parameters in red and the initial conditions in blue at the top of the diagram.

In Fig. 8 we show the dynamical loop that integrates the dynamical equations with an Euler method. The program is initiated by choosing the values of the number of cells (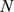), the position of each cell (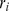), their proliferation rate (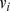), the gates given by the PIN action between two cells (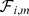) and the concentration of auxins (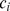) at time 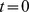.

It is important to note that we normalize the auxin concentration function 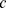 with its maximum value at every time step. This allows our model to take into account the role of possible sources and sinks of auxin, since 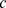 is not a conserved quantity. The final distribution of auxin is insensitive to the initial conditions, but we start with a random distribution of auxin with a maximum at the quiescent centre in accordance with experimental observations. We recovered the same results if auxin concentrations were random at initial conditions (data not shown). The cycle clocks of each cell are set to zero at 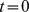 and reset after a successful cell division.

The shape and color of the boxes (Fig. 8) represent the action of the different dynamics as described in experimental systems (see Fig. 3). The red square indicates a subroutine that includes the logical rules 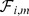 of the PIN action and the red circles represent points of logical decisions at appropriate times. Black arrows represent the direction of flux of the simulation and the black-dash arrow indicates a decision related to the time condition for the dynamics of the cyclins. Eq. 4 is implemented in the blue diamond block that represents the elastic field with time scale 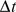. The loop is performed while the time 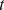 is less than the final time 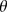. Eq. 7 is implemented in the green block. The cyclin period is calculated for each cell at the violet block using Eqs. 8 and 9 and the threshold 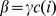. Cellular divisions are performed as a subroutine represented by the orange block, and cell proliferation alters the conditions of all three dynamics.

The first step is to estimate the values of the parameters of the system. The adjustable parameters are the quantities indicated in red in Fig. 8. We start with the kinematical parameters. The constant 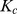 is related to the elastic modulus 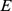 of the cells. This quantity is measured when studying the mechanics of walls, cells, and tissues and is of the order of 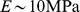, as reported in [67].

For simplicity let us consider hexagonal cells in equilibrium. The magnitude of the elastic force is 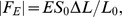 where 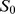 is the contact area between two cells, 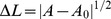 is the change in length just after division, where 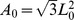 is the area of the hexagon, if 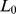 is the distance between centroids of two contiguous cells. This should be equal to the corresponding force magnitude in our model 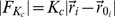. Just after a cell division, 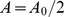, thus 
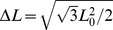
, 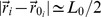 and 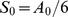. Equating the two forces we obtain
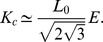
Taking the average diameter of a mature cell as 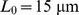 [64] and the experimental value 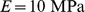 we obtain 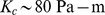.

At this stage, 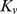 should be related to the properties of the cell membrane, the metabolism of cell growth and the turgor pressure. It is difficult to associate the action of the first term of Eq.(1) to a single biological property. However, the dynamics of this term should produce a restoring force of the same order of magnitude as the second term, if the form and geometry of the domain are to be maintained during the growth dynamics. Therefore, if we use a value of 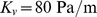 the system should relax to a set of cells with roughly the same size and shape, as shown in the calculation of Fig. 5B. We found numerically that this produces results for the dynamics of growth that are comparable to the experimental quantities measured.

Parameter 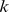 is related to the viscous damping of the cell motion. The dynamical friction constant 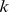 can be estimated by observing that the amplitude of the oscillatory motion 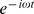, caused by the harmonic forces should be reduced, to avoid oscillations, by a factor of 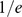 in a lapse of at most one period 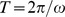, that is 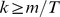. Note that in Eq. (4) the mass of the cell (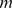) is considered to be one. This gives 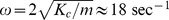, and 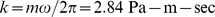.

The values used in the calculations are 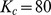, 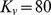 and 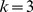. With these values we obtain the real time scale of an iteration step 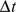 in the numerical calculations, by finding the number of iterations needed to obtain the experimental number of cell divisions in that lapse. In seven days, our observations showed (see Fig. 1) that the number of cells in the meristem is about 350. In averaged calculations we reproduce this number in 3400 iterations by using 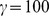 and 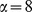. This means that the lapse representing one iteration is the number of minutes in 7 days over the number of iterations, that is 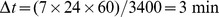. Considering that the average auxin concentration is 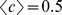, the value of 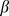 is 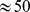 in units of 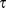, which is about 100 times 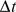. These values produce a single cell cycle period on the order of 12 hr, as shown in Fig. 7 [66].

**Figure 9:**
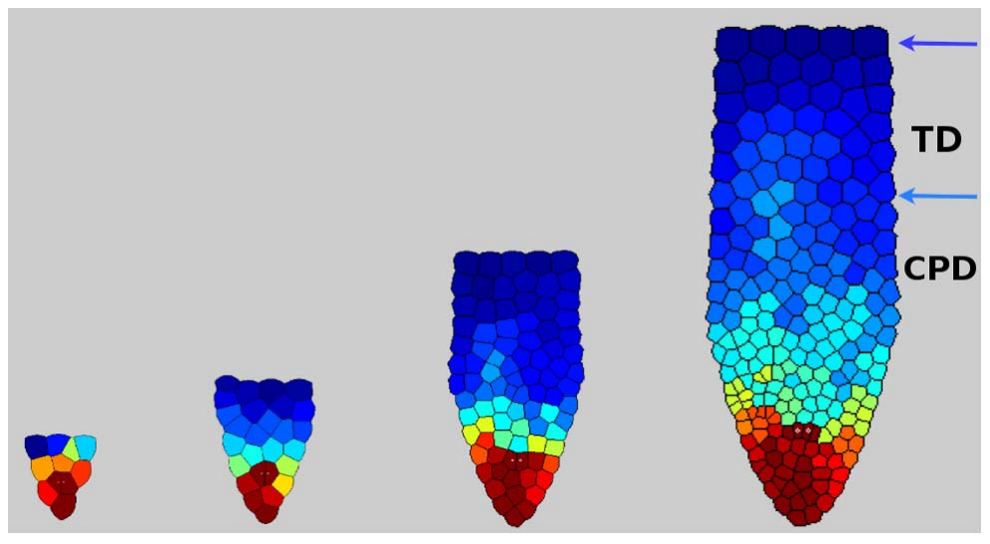
Typical calculation of the dynamical growth of the root using the model described. We show four snapshots of the configuration at 400, 1400, 2400 and 3400 time steps. The color code represents the concentration of auxins, red for the maximum and blue for the minimum. See Video S5.

In Fig. 9 (and Video S5) we provide an example of the growth of the system. We start with eight points at random in the parabolic tip of the domain, and fix the position of two additional points that represent the quiescent cells in the centre of the domain, marked with a white symbol. These cells reproduce at a rate ten times lower than the others; they divide after ten divisions per cell on average (in the right panel of the figure these quiescent cells have just divided). The auxin concentration in these cells is set to the maximum initially, and this is represented by a dark red color in the figure. The cell's position, shape, and proliferation rate are calculated every time step and the auxin is transported between cells. After 400 time steps the cells are attaining a uniform shape and size (Fig. 9), and the auxin gradient is already formed. This gradient will dictate the time in which a complete cell proliferation cycle is accomplished locally, followed by a cell division event that produces a sudden increase of the local potential that, in turn, governs auxin transport.

**Figure 10:**
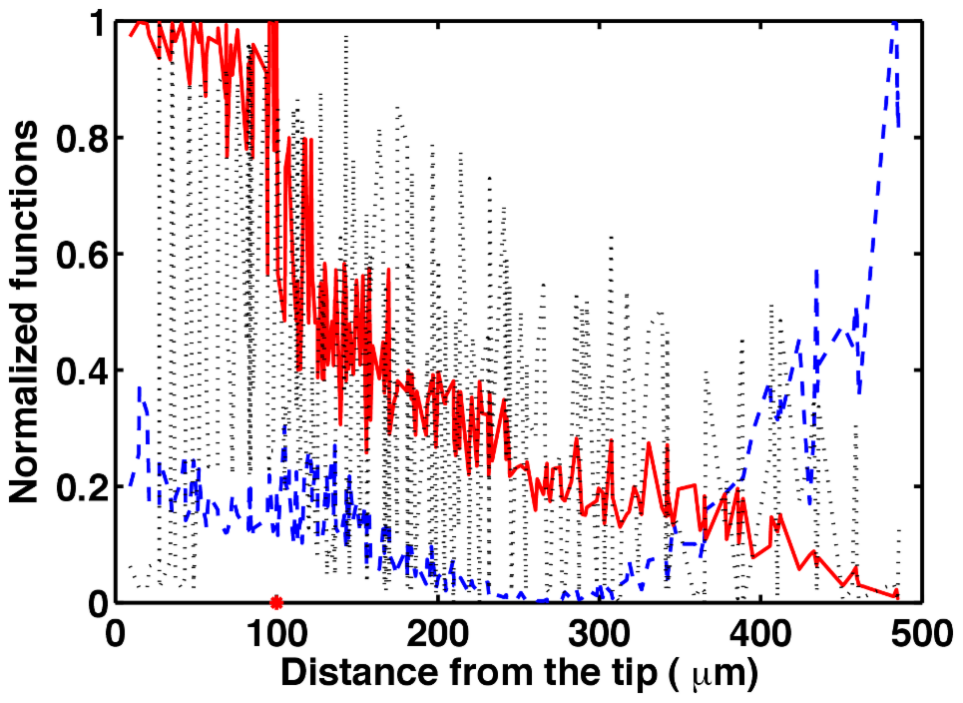
Plots of local potential, auxin concentration and cell cycle, after coupling dynamics. The normalized local potential (dashed-blue), the auxin concentration (red) and the advance of the cycle clock (dotted-black) as functions of the distance from the tip (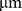), at 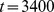 time steps, corresponding to seven days.

Despite these complicated dynamical interactions, the auxin gradient is preserved throughout and the process of growth and cell patterning is by no means random. This can be seen in Fig. 10. The overall pattern that emerges after some cycles of coupled dynamics is very similar to the apical-basal pattern of cell proliferation and elongation observed in RAM and along the length of the root tip. Such dynamics and emergent pattern are robust to initial conditions.

It is interesting to note that the region around the quiescent center in the stem-cell niche shows the greatest concentration of auxin, and a maximum in the potential. Also, the cell division cycle is minimum at this location. An intermediate region in which the auxin concentration diminishes and the potential is very small, but the cell proliferation rate is roughly constant, surrounds the quiescent cells. Finally, the most distal part from the tip (towards the base of the plant) is characterized by a very small concentration of auxin, causing the cell proliferation rate to be very small, and the potential to increase enormously. The combination of these effects results in the arrest of cell proliferation and in the formation of the elongation zone at a defined distance from the root tip. The emergent patterns recovered in the model are similar to those observed for the distribution of auxin as reported in Ref. [68], and the pattern of cell proliferation along the root longitudinal axis reported in Ref. [69]. Our results are also in agreement with the qualitative patterns of cell proliferation and elongation that are observed along the apical-basal, longitudinal axis of the growing *A. thaliana* root.

**Figure 11:**
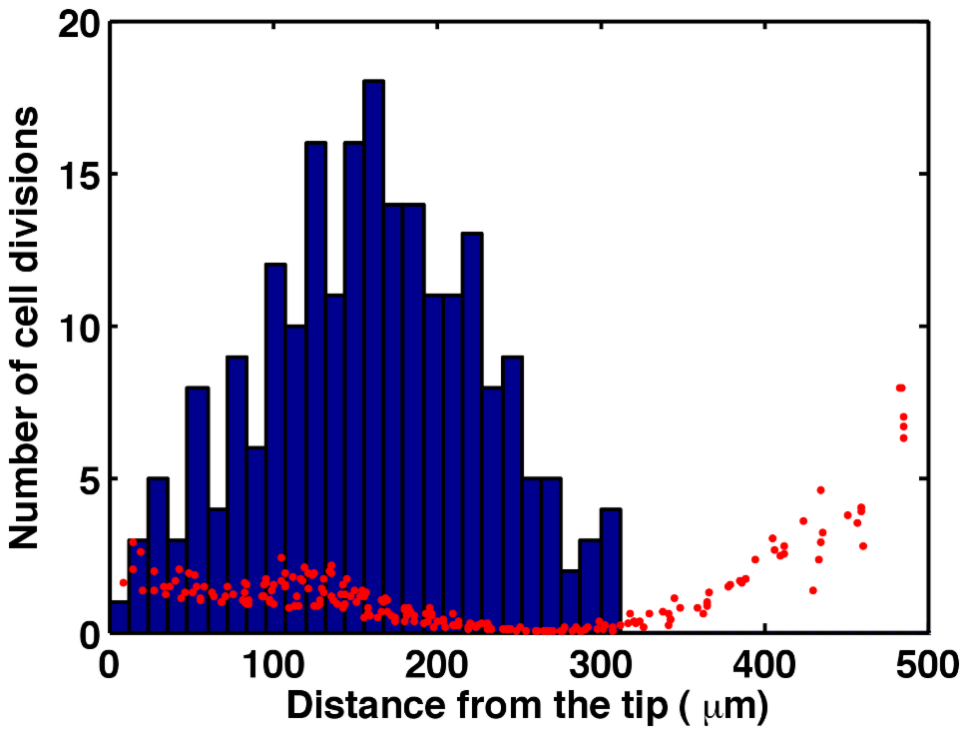
Histogram of the number of cell divisions obtained along the root when 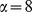
 **.** The potential profile is shown as red dots. Observe that there are no cell divisions beyond 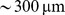, meaning that the meristem has attained a stationary length.

We can use this model to predict what patterns are expected under different growth conditions. In Fig. 11 we show a histogram of the number of cell divisions occurring at a given distance from tip, as obtained from an example calculation in which we fixed the parameter 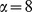. Interestingly, we observe that the length of the RAM does not surpass a certain value, which depends on 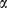, because the modeled coupled dynamics prevents cells far from the tip to divide. Such types of coupled dynamics could explain the emergence of the transition from proliferation to the elongation cellular states in real roots, as well as the limited ranges or domain sizes of actively proliferating cells in stem-cell niches of plants and animals [1], [3].

Hence, our model can be used to generate novel predictions concerning the role of the parameters considered in the model, and in determining RAM size and cell proliferation and elongation patterns along the root apical-basal axis for *A. thaliana* under different environmental or growth conditions. Our general model could eventually be adjusted to model stem-cell niches in other plants and animal systems, as well as modeling growth and differentiation in communities of unicellular organisms if similar physical fields can be postulated in such latter cases.

**Figure 12:**
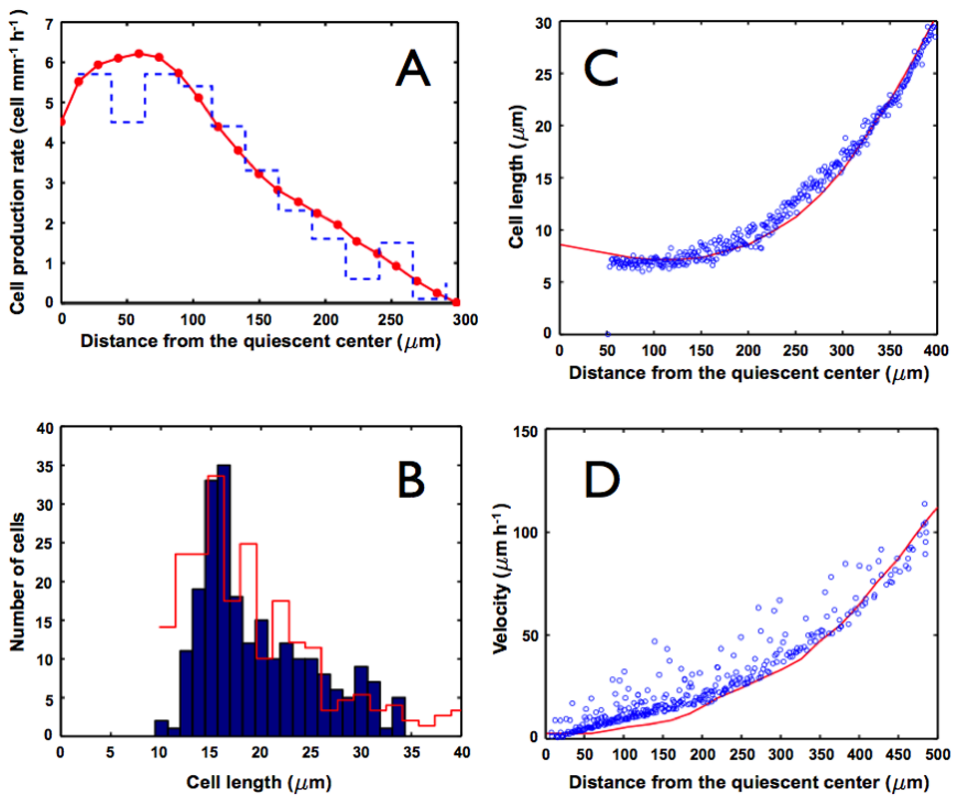
Comparisons between results obtained with the model and experimental data. (A) Cell proliferation rate as a function of the distance from the quiescent centre; calculation from Fig. 9 after six days of growth. The red line and dots are the experimental points reported in Ref. [64]. (B) Frequency distribution for cell length. Experimental data were taken from our laser microscope image of Fig. 1. (C) Average cell length as a function of the distance from the quiescent centre; calculation from Fig. 9 after six days of growth. The red line is the experimental result reported in Ref. [64]. (D) Average cell proliferation velocity as a function of the distance from the quiescent centre; calculation from Fig. 9 after six days of growth. The red line is the experimental result reported in Ref. [64].

In order to examine the quantitative behavior of the model and validate it with published experimental data, we compared our model's predictions to measurements of the proliferation rates along the axis of the *A. thaliana* root as a function of the distance from the quiescent centre [64]. We ran numerous iterations of the model in order to obtain a reasonable statistical sample. We show a typical result from the simulations run to the experimental data in Fig. 12. Panel (A) shows the available experimental results for cell proliferation rates along the apical-basal axis of the root reported in Ref. [64] as a continuous red curve. The numerical results from our model are shown in blue. These results were obtained using the estimated parameter values that give the time in hours and the sizes in 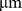. We shifted the origin to account for the fact that all quantities in our calculations were measured from the tip of the domain and not from the quiescent centre. Notice that the simulated and experimentally generated curves are very similar.

In Fig. 12(B) we show an histogram of the frequency distribution of cell size. This histogram varies with different iterations because of the stochastic nature of cell proliferation and growth dynamics [70]. However, all calculations share the same qualitative characteristics; namely an unimodal distribution between 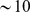 and 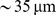, with a maximum around 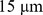. This result was already recovered by Verbelen and collaborators Ref. [71]. The red curve was obtained by measuring the cell size in an Arabidopsis root Fig. 1. Similar curves have been obtained for many different plant species, including wheat [70].

In Fig. 12(C) we show the variation of cell length along the longitudinal axis of the root. The red curve is the experimental result from Ref. [64]. It should be pointed out that the experiment was obtained by measuring the cell flux in a fixed point and by counting along the axis of the root in two dimensions, which is very convenient when comparing with our two-dimensional model. In order to mimic the experimental procedures, our numerical results were obtained by spotting the centroids of the Voronoi cells in the final time, which corresponds to six days. We calculated the length (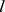) by assigning an area of 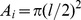 to each cell. Again, a shift of 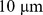 in the horizontal axis was needed to account for the difference in the origin, and the results for each cell are displayed as blue dots in the figure. Once again, the agreement between our simulated results and the experimental data are clear.

Finally, in Fig. 12(D) we show the average cell proliferation velocity, as defined in Ref. [64], as a function of the distance from the quiescent centre (red line), and compare it with our results (blue dots). In the experiment, Beemster and collaborators measured the difference in position of each cell for two subsequent times, averaged over time. In our calculation we measured the difference in position of each cell with respect to the apex of the root, which is itself being displaced every time a cell division takes place. By changing the frame of reference, we can compare the reported experiment with our results. The agreement is also remarkable when one compares the simulation results recovered with our model and the experimental data. This is more significant than the previous validations, since this result reflects the totality of the dynamical behavior in time and not only in a frozen snapshot, as in the previous cases.

## Discussion

We present a dynamical model that couples auxin concentration gradients, cell proliferation and a physical tension field in a two-dimensional spatial domain that mimics the *A. thaliana* root tip. We have validated our model with both static and dynamic cellular empirical data, and have shown that our model recovers the pattern of rates of cell proliferation observed in the apical-basal axis of roots. The model also recovers the discrete transition from the proliferative to the elongation domains. Thus, our model puts forward a novel theoretical framework to test hypothesis concerning the coupled roles of auxin, cell proliferation, and physical fields dynamics in the emergence of the cellular pattern observed along the *A. thaliana* root tip. Ultimately, we have postulated a complex system in which the main emergent property of the coupled dynamics is at the appropriate spatial and cellular structure for the intracellular genetic networks to express differentially along the root. However, the explicit consideration of complex gene regulatory networks is out of the scope of this paper.

**Figure 13:**
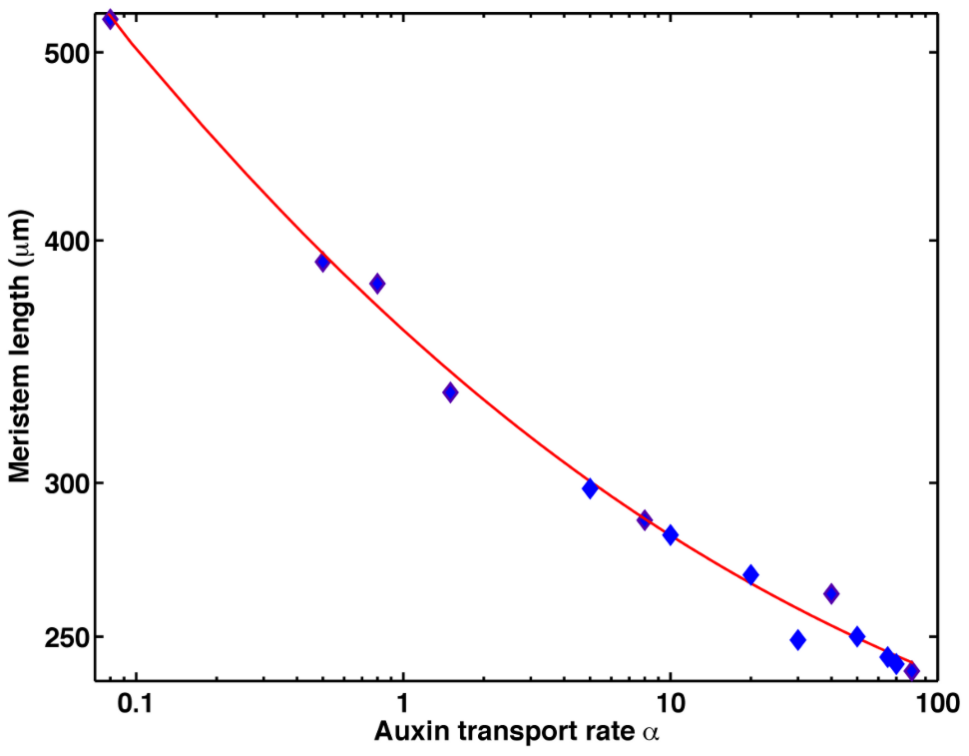
Log-log plot of the maximum RAM as a function of the parameter 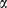. Numerical results are blue rhombuses, and the red line is the best fit with a function of the form 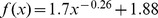 with R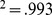.

Our model and analysis suggest that the size of the RAM depends on the value of the parameter 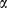 (Fig. 13) in a rather defined manner. This parameter represents the ratio between the time scales of the potential relaxation and the auxin transport mechanisms. The length of the RAM decreases as the auxin transport parameter 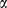 increases as a power law. Therefore, this quantitative prediction can be verified experimentally, as auxin concentrations and transport along the root can be modified by manipulating the conditions of root growth (e.g. adding NPA to the growth medium to block auxin transport). Previous experimental work has suggested that the size of the RAM varies depending on growth conditions and is altered with external supplementation of auxin [39].

Given that plant growth is influenced by the mechanical behavior of the cell wall, measurements of the mechanical properties of living cell walls are important to fully understand how cellular organization is achieved. Like most biological materials, material properties of cell walls change as a function of age, the magnitude of forces they are subjected to, and immediate physiological conditions [67], [72]. This confers spatial and temporal heterogeneity on cell wall constituents, complicating measurements of the mechanical properties of plant living walls even with present-day instrumentation. Furthermore, a single modulus of elasticity is not sufficient because of the structural anisotropy of the cell wall [67]. Therefore, comparisons between the predicted values of Kv and Kc of our model and the values reported for the modulus of elasticity of real cell walls are far from being straightforward. However, the fact that we reproduced the root tip pattern with the selected values suggests that they are likely to be biologically meaningful.

More generally, our work reinforces conclusions from recent studies that experimentally demonstrate the importance of physical forces in the regulation of root apical-basal patterning [49], [53], such as the mechanical induction of lateral roots or the coordination between auxin concentration and microtubule orientation [49], [53], [54]. It is remarkable that simple arguments concerning uniform size, shape and geometry of cell disposition is sufficient to produce a non-uniform field that provides sufficient spatial information to recover the overall dynamical growth pattern observed along the root. It is thus predicted that modification of physical forces would change the size and the pattern of these zones, an issue that could in principle be further explored theoretically and experimentally.

Auxin response is modulated not only by auxin concentration, but also by the auxin signaling pathway, which includes many components of different gene families, and which interact through several feedback loops, creating non-linear behaviors. Consequently, auxin concentration at any location does not necessarily coincide with auxin response. Even if this is not the case in the root [68], it could be important to include an explicit model of the auxin signaling pathway in future extensions of our model. In addition, in our model we considered the polar PIN configuration as fixed, as in Ref. [46]. However, in reality a more robust dynamic auxin transport is observed when the PIN expression is regulated by auxin [73].

In our model we fixed the position and number of the quiescent cells. We are aware that the root stem-cell niches are regulated by a complex regulatory network [74]. WUSCHEL RELATED HOMEOBOX5 (WOX5) is a Quiescent Center identity gene indispensable for the maintenance of the undifferentiated state of stem cells and niche size regulation, and it is part of the proposed root stem-cell niche regulatory network [74], [75]. Recent theoretical and experimental work has suggested that WOX5 regulates and is regulated by auxin [36], [74]. In our calculations we input several initial conditions for auxin concentration, and demonstrated that the model is fairly robust to these changes. However, as shown in Fig. 6, neglecting the action of PIN polarization destroys the auxin gradient along the root. Including these and other regulatory interactions in a future model would enable us to explicitly consider intracellular complex gene regulatory networks, which are likely coupled among cells by physical and hormone fields.

The complex network underlying the cell cycle was also reduced to consider two basic components, because for our purposes, only the phases of the oscillations of the concentrations matter. Since CYCA and CYCB oscillate in phase, we consider them as a single variable; and because CYCD oscillates in anti phase, we take this to mean that there is an activator-inhibitor interaction between these two groups of proteins. In our model we stressed the importance of the relationship between auxin concentration and the regulation of cell proliferation, and we neglected the details of the known regulatory processes of the cell cycle, which although important, do not directly affect the overall results of our simulation. Nonetheless, such details of the gene regulatory network underlying the cell cycle, cell differentiation and auxin dynamics should be incorporated in future developments of the model.

In conclusion, we have put forward a minimal mathematical model that considers the essential dynamical coupling of cell proliferation with a physical field and chemical (hormone) gradients, in order to explore if such processes are sufficient to obtain the emergence of cellular organization during stem-cell niche patterning and organ growth. We have used the *A. thaliana* root as our study system.

Despite the simplification of many biological details, our model is able to recover patterns that greatly resemble those observed in stem-cell niches of plants and animals, and particularly those in the *A. thaliana* root tip. The remarkable coincidence between the simulated cellular characteristics along the model root apical-basal axis (shown in Fig. 12), with those that have been observed and quantified in actual roots, validates the qualitative features and utility of our model for understanding the emergence of cellular patterns in such a multicellular organ. Furthermore, the cellular patterns of stem-cells among multicellular plants and animals have generic traits. Our model provides a formal tool to explore if such traits may be explained by the generic non-linear coupling of relevant physical and chemical fields to discover emergent properties of cell proliferation dynamics across biological systems.

## Supporting Information

Video S1 **Dynamical growth and proliferation of cells and boundary.**
(MP4)

Video S2 **Dynamical behavior of stem cells only.**
(MP4)

Video S3 **Relaxation process for a fixed number of cells.**
(MP4)

Video S4 **Dynamical changes of the local cell potential.**
(MP4)

Video S5 **Dynamical development of auxin concentration.**
(MP4)
